# Comparison of intima-media thickness of common and internal carotid arteries of patients with ischemic stroke and intracerebral hemorrhage

**Published:** 2014-10-06

**Authors:** Ali Moghtaderi, Sharareh Sanei-Sistani, Ghassem Abdollahi, Hamid Dahmardeh

**Affiliations:** 1 Department of Neurology, School of Medicine, Zahedan University of Medical Sciences, Zahedan, Iran; 2 Department of Radiology, School of Medicine, Zahedan University of Medical Sciences, Zahedan, Iran

**Keywords:** Intima-Media Thickness, Common Carotid Artery, Internal Carotid Artery, Ischemic Infarction, Intracerebral Hemorrhage

## Abstract

**Background: **Role of atherosclerosis in the pathogenesis of ischemic and hemorrhagic infarctions is still matter of debate. Intima-media thickness (IMT) of the common carotid artery (CCA) and internal carotid artery (ICA) are markers of atherosclerosis. The aim of this study was to compare CCA IMT and ICA IMT of patients with ischemic and hemorrhagic infarction.

**Methods:** Two equal groups of 80 patients with small and large vessel ischemic stroke and 80 patients with non-traumatic intracerebral hemorrhage (ICH) who referred to our central teaching hospital of Zahedan were assessed in this descriptive study. IMT of four arteries (right and left CCA and ICA) were measured, and collected data were analysis using Student’s t-test.

**Results: **There were 137 males (57.1%) and 103 (42.9%) female with mean age of 62.7 ± 11.7. Mean right CCA IMT of patients with small vessel diseases (SVD), large vessel diseases (LVD), and ICH were 0.564 ± 0.130, 0.623 ± 0.150, and 0.580 ± 0.140 mm, respectively (P = 0.032). Mean left CCA IMT of patients with SVD, LVD, and ICH were 0.569 ± 0.120, 0.618 ± 0.120, and 0.573 ± 0.130 mm, respectively (P = 0.039). The above findings for right ICA were 0.572 ± 0.120, 0.569 ± 0.140, and 0.522 ± 0.130 mm, respectively (P = 0.145). Those findings for left ICA IMT were 0.525 ± 0.110, 0.554 ± 0.120, and 0.527 ± 0.120 mm, respectively (P = 0.257).

**Conclusion: **Our findings showed that by using CCA IMT, differentiation between small and large vessel infarctions and hemorrhagic infarctions can be made.

## Introduction

Atherosclerosis is increasingly recognized as an inflammatory disease and stroke are the leading cause of disability worldwide. It is introduced as the third most common cause of death beside disability in the world by some studies. The term stroke describes a vascular event which is associated with a sudden appearance of neurological symptoms. Strokes usually occur in the elderly patients and its treatment costs are too much for their families and the insurance companies. Patients are prone to develop many kinds of complications such as pneumonia, urinary tract infections, pulmonary embolism, deep vein thrombosis, and pressure ulcers, which any of them could lead to morbidity.^1^^,^^2^

Main symptoms and signs are unilateral weakness, paralysis, numbness and loss of sensation of upper and/or lower extremities. Aphasia, visual field impairment and ataxia are also other symptoms of a stroke.^3^^,^^4^ Approximately, 85% of vascular insults of the brain are ischemic in origin, and the rest are primary cerebral hemorrhage such as subarachnoid and intra-parenchymal hemorrhage. Arterial hypertension (HTN), diabetes mellitus (DM), obesity, hypercholesterolemia, and smoking are the major risk factors of different types of cerevrovascular accidents.^5^

One of the most important predisposing factors for stroke is atherosclerosis of carotid artery that plays an important role in the mechanism in more than 1/3 of stroke patients. Many types of heart diseases such as valvular diseases, dysrhythmias, and intracardiac right to left shunts prone the patients to embolic stroke. Large vessel diseases (LVD) are due to atherosclerosis of large arteries, cardiac embolism, and other uncommon reasons; however, small vessel diseases (SVD) or lacunar infarctions are due to occlusion of small cerebral arteries,^6^^-^^8^ that is commonly the result of lipohyalinosis of arteriols.^1^

Current clinical guidelines recommend additional diagnostic tests to assess vascular problem before or after any vascular insult in the brain. Duplex ultrasound study of the carotid arteries is recently used for assessing wall thickness and smoothness of the cerebral vasculature. One diagnostic method of assessing atherosclerosis measures the carotid artery intima-media thickness (IMT). It is a marker for atherosclerosis and may be used as a risk factor for stroke. B-mode ultrasonography is a non-invasive method for measuring this thickness. IMT measurement has been extensively used in population based studies. Intima-media are a complex of endothelial cells, connective tissue, and myocytes, which can be a place for lipids to accumulate and form atherosclerotic plaques. Thus, thickened intima-media of carotid artery can be an indicator of generalized atherosclerosis.^9^^,^^10^ Regarding the IMT seems to be an enhancing factor of atherosclerosis and findings of different studies are controversial, the main objective of this study was to compare carotid artery IMT in patients with small vessel ischemic infarctions (SVD), large vessel ischemic infarctions (LVD), and patients with intracerebral hemorrhage (ICH) with each other in Imam Ali Teaching Hospital in Zahedan, Iran.

## Materials and Methods

In this study, totally 240 patients with SVD, LVD, and ICH in three equal groups of 80 were entered into the study. IMT of four arteries [common carotid artery (CCA) and internal carotid artery (ICA) on the right and left sides] were measured. Patients selected according to the World Health Organization (WHO) definition. Based on WHO, stroke is defined as “acute focal or global disturbance of cerebral function lasting 24 h or more or symptoms lasting less than 24 h if a brain imaging study showed an ischemic lesion appropriate to the symptoms due to a cerebral vascular insufficiency.”^11^ A study was approved by the Ethics Committee of the Zahedan University dean for research affairs. Informed consent was obtained before performing the procedure for each patient. Later, the examiner filled out a questionnaire about participants’ age, gender, occupation and other associated disorder related to their stroke. For patients with stroke symptoms, magnetic resonance imaging or computed tomography scan of the brain was performed at the emergency department in order to choose proper management. Patients were classified to three groups of SVD, LVD, and ICH according to the imaging findings. Other underlying factors were defined according to standard definition as a disease or behavior.

A patient was considered to have DM who had fasting plasma glucose more than 126 mg/dl twice, or random plasma glucose more than 200 mg/dl plus classic diabetes symptoms or who was a known case of diabetes and was treated with insulin and/or other glucose-lowering agents.

A patient was considered to have HTN, who had systolic blood pressure higher or equal to 140 mmHg or diastolic blood pressure above 90 mmHg measured at standard conditions, or who was known case of HTN and was treated with antihypertensive drugs. Hypercholesterolemia: A patient was considered to have hypercholesterolemia whose blood cholesterol level was above 240 mg/dl or who was known case of the disease and was treated with cholesterol-lowering agents. Smoking: a patient was considered to be a smoker who had a positive history of smoking of more than 10 years.

After completing questionnaire information, all patients underwent high-resolution real-time sonography using (GE Logic 500 ultrasound system; GE Medical System, USA) with 5-7 MHz linear array transducer. The IMT of bilateral common and internal carotid arteries of each group of patients was measured using. The procedure was described for the patients or their guardians in case of defect in their consciousness. Two radiologists (HD and GA) with practical experience in this issue who was blinded to clinical findings performed all examinations. Patients lying in the supine position and their necks were extended. Carotid arteries 2 cm below and 2 cm above the carotid bifurcation were examined, and IMTs of those parts were measured. The ultrasound machine had software for automatic measuring and calculation of the IMT, but it was reevaluated with the examiner and in case of any inconsistencies the examiner check it again and measure it manually.

The authors used SPSS for Windows 20.0 (SPSS Inc., Chicago, IL, USA) arithmetic mean, and standard deviations of different variables were calculated. Chi-squared and t-tests were applied to compare the continuous and dichotomous variables between cases and control groups respectively.

## Results

Mean age of patient in SVD, LVD, and ICH were respectively 63.5 ± 11.8, 63.7 ± 10.9, and 60.2 ± 12.5 years (P = 0.162). There was also no difference among three groups for gender distribution (P = 0.095). More than half of patients (55.0%) had HTN, 77 patients (32.1%) had DM, 56 patients (23.3%) hypercholesterolemia and 22 patients (9.2%) were smoker (P < 0.050) (Table 1).

Mean right CCA IMT of patients in SVD, LVD, and ICH groups were 0.564 ± 0.130, 0.623 ± 0.150, and 0.580 ± 0.140 mm, respectively (P = 0.032). Mean left CCA IMT of patients in SVD, LVD, and ICH groups were 0.569 ± 0.120, 0.618 ± 0.120, and 0.573 ± 0.130 mm, respectively (P = 0.039). There was a significant statistical difference between mean CCA IMT on right and left sides (P = 0.020) (Table 2).

Mean right ICA IMT of patients in SVD, LVD, and ICH groups were 0.572 ± 0.120, 0.569 ± 0.140, and 0.522 ± 0.130 mm, respectively (P = 0.145). Mean left ICA IMT of patients in SVD, LVD, and ICH groups were 0.525 ± 0.110, 0.554 ± 0.120, and 0.527 ± 0.120 mm, respectively (P = 0.257). Furthermore, there was no significant statistical difference between mean IMT of ICA of right and left sides (P = 0.151) (Table 3). 

**Table 1:** Frequency of risk factors in three groups

**Factor**	**Groups**				**P**
	**SVD, n (%)**	**LVD, n (%)**	**ICH, n (%)**	**Total, n (%)**	
Diabetes mellitus	23 (28.8)	30 (37.5)	24 (30.0)	77 (32.1)	0.439
Hypertension	39 (48.8)	46 (57.5)	47 (58.8)	132 (55.0)	0.383
Hypercholesterolemia	18 (22.5)	23 (28.8)	15 (18.8)	56 (23.3)	0.319
Smoking	7 (8.8)	11 (13.8)	4 (5.0)	22 (9.2)	0.319

**Table 2:** Comparison of intima-media thickness of right and left common carotid arteries among three groups of small vessel disease, large vessel disease, and intracerebral hemorrhage

**Common carotid artery**	**Group**	**Mean ± standard deviation**	**P**
Right	SVD	0.564 ± 0.130	0.032
	LVD	0.623 ± 0.150	
	ICH	0.580 ± 0.140	
Left	SVD	0.569 ± 0.120	0.039
	LVD	0.618 ± 0.120	
	ICH	0.573 ± 0.130	
Mean of right and left	SVD	0.566 ± 0.120	0.020
	LVD	0.620 ± 0.130	
	ICH	0.516 ± 0.130	

SVD: Small vessel disease; LVD: Large vessel disease; ICH: Intracerebral hemorrhage

**Table 3:** Comparison of intima-media thickness of right and left internal carotid artery among three groups of small vessel disease, large vessel disease, and intracerebral hemorrhage

**Internal carotid artery**	**Group**	**Mean ± standard deviation**	**P**
Right	SVD	0.572 ± 0.120	0.145
	LVD	0.569 ± 0.140	
	ICH	0.522 ± 0.130	
Left	SVD	0.525 ± 0.110	0.257
	LVD	0.554 ± 0.120	
	ICH	0.527 ± 0.120	
Mean of right and left	SVD	0.526 ± 0.110	0.151
	LVD	0.561 ± 0.120	
	ICH	0.519 ± 0.120	

SVD: Small vessel disease; LVD: Large vessel disease; ICH: Intracerebral hemorrhage

## Discussion

Quantitative evaluation of atherosclerosis by ultrasound was first described by Eugene at early 1980s.^12^ Pignoli^13^ and Pignoli et al.^14^ then described measurement of IMT and its accordance with pathology. He and his colleagues showed that there is not any significant difference between B-mode measurement of IMT in vivo and in vitro. They concluded that using ultrasound is safe and accurate for this kind of measurement. Since then IMT has become an appropriate mean for evaluation of atherosclerosis. We also used IMT to compare patient with SVD, LVD and ICH and found mean CCA IMT of LVD patients to be significantly more than SVD and HI patients.^15^

Pruissen et al.^16^ compared CCA IMT between SVD and LVD patients. They found that CCA IMT is thicker in LVD patients than in SVD patients supporting the hypothesis that LVD and SVD have a different pathogenesis, and more or less it is the same as our results. They classified 417 of their patients as LVD and the remained 115 as SVD. Finally, their report showed intima media of CCA of LVD stroke patients is thicker than patients with lacunar infarctions. Cupini et al.^17^ also indicated the usefulness of non-invasive measurement of IMT with ultrasonic techniques as a diagnostic tool that may help to identify different subtypes of ischemic stroke patients. There may be a predictive power with respect to lacunar versus non-lacunar infarcts. They also concluded that the higher mean for CCA IMT of patients with non-lacunar infarctions compared with patients with lacunar infarctions and control group. Study of Freitas et al.^9^ among three groups of patients with ischemic stroke, hemorrhagic stroke and no event showed that with the increase of CCA IMT risk of ischemic stroke increases; therefore they introduced increased CCA IMT as an independent predictor of the risk for ischemic stroke, but not for hemorrhagic stroke.

In a study for comparison of CCA IMT of stroke patients and patients admitted due to reason other than stroke by Touboul et al.^18^ Mean measurements for CCA IMT of 470 stroke patients and 463 control groups were 0.797 and 0.735 mm, respectively. They also reported meaningful relationship between stroke and increased IMT. Tsivgoulis et al.^19^ found increased CCA IMT is associated with long-term increased risk of stroke recurrence in their selected patients. Ohira et al.^20^ also reported a close relationship between CCA IMT and hemorrhagic and ischemic strokes; moreover, the relationship between IMT and lacunar infarctions was confined to African-American, and it does not have the same strength in whites. In their study, the estimated risk ratios of carotid intima-media thickening for stroke subtypes were higher for cardio-embolic and non-lacunar strokes than for hemorrhagic and lacunar strokes. They have also shown that the incidence rates of hemorrhagic and ischemic strokes were greater across higher carotid IMT levels. 

However, there are some inconsistencies between the results of different research published studies. Some of them invalidate the appropriateness of IMT. Lorenz et al.^21^ reported CCA IMT as an independent factor in vascular events and after a follow up of 4.2 years they concluded that its predictive value was the same in young and old patients. Ellul et al.^5^ reported that CCA IMT has no relationship with adverse functional outcome after ischemic stroke.

In the present study, patients of ICH group had a lower mean age, which is similar to study of Khorvash et al.,^22^ Khan and Vohra,^23^ Lin et al.,^24^ and Sadeq.^25^ In patients with ischemic and ICH, there is a high prevalence of diabetes mellitus, hypertension, hypercholesterolemia, and smoking. In our study, prevalence of HTN was slightly higher in the hemorrhagic stroke group than in ischemic stroke. Inversely diabetes mellitus, hypercholesterolemia, and smoking were more prevalent among ischemic stroke patients, which is consistent with results of studies of Khorvash et al.^22^ and Sadeq.^25^ It should be declared that HTN was the most frequent and one the most important risk factors in our study. We believe that there is a statistical difference between mean IMT thickness of patients with ischemic stroke and cerebral hemorrhage in common carotid arteries. Probably, HTN may act as potential risk factor in those arteries in ICH patients.

It should be noted that there was no significant relationship between ICA IMT among two Groups, but study of Kitamura et al.^26^ showed that the combination of the CCA and ICA IMT results in better prediction of stroke than CCA IMT alone.

## Conclusion

This study showed that there is a statistical difference between the mean measured values for CCA IMT of large vessel, small vessel and hemorrhagic vascular brain insults, and it may be useful in the differentiation between different kinds of ischemic infarctions and hemorrhagic infarctions. More studies with a high number of patients may be required for the out coming studies.

## Notes

**How to cite this article: **Moghtaderi A, Sanei-Sistani Sh, Abdollahi Gh, Dahmardeh H. Comparison of intima-media thickness of common and internal carotid arteries of patients with ischemic stroke and intracerebral hemorrhage. Iran J Neurol 2014; 13(4): 226-30.
